# The Role of Inflammatory Biomarkers in PIPAC: Predicting Survival and Treatment Completion in Patients with Peritoneal Metastasis

**DOI:** 10.7150/jca.123687

**Published:** 2026-01-01

**Authors:** Signe Roensholdt, Martin Graversen, Sönke Detlefsen, Claus Fristrup, Per Pfeiffer, Michael Bau Mortensen

**Affiliations:** 1 Odense PIPAC Center, Odense University Hospital, 5000 Odense, Denmark.; 2 Department of Clinical Research, Faculty of Health Sciences, University of Southern Denmark, 5230 Odense, Denmark.; 3 Department of Surgery, Odense University Hospital, 5000 Odense, Denmark.; 4 Department of Pathology, Odense University Hospital, 5000 Odense, Denmark.; 5 Department of Clinical Oncology, Odense University Hospital, 5000 Odense, Denmark.; 6 Open Patient Data Explorative Network, Region of Southern Denmark, 5000 Odense, Denmark.

**Keywords:** advanced cancer, peritoneal metastasis, PIPAC, inflammatory biomarkers, prognostics

## Abstract

**Introduction:** Appropriate patient selection is essential for optimising outcomes in individuals with peritoneal metastasis (PM) undergoing treatment with Pressurized Intraperitoneal Aerosol Chemotherapy (PIPAC).

This study investigated the prognostic value of pretreatment inflammatory biomarkers and explored their ability to predict the possibility of completion of three or more PIPAC treatments.

**Method:** This observational study analysed prospectively collected data from patients with PM of gastrointestinal or ovarian origin enrolled in the PIPAC OPC-1 or OPC-2 studies between March 2015 and January 2022. Six biomarkers were examined: Neutrophil-to-Lymphocyte Ratio (NLR), Platelet-to-Lymphocyte Ratio, Systemic Immune-Inflammation Index (SII), C-reactive protein, modified Glasgow Prognostic Score, and Prognostic Nutritional Index. Biomarkers were obtained pretreatment, and treated as continuous variables. Survival was assessed using Kaplan-Meier and Cox regression analyses, adjusting for covariates available prior to the first PIPAC. ROC analysis was used to evaluate the predictive performance. A p-value less than 0.05 was considered statistically significant.

**Results:** The cohort consisted of 130 patients, with a median overall survival (OS) of 8.7 months. Sixty percent of the patients received three or more PIPAC treatments. Elevated levels of all six biomarkers were significantly associated with reduced OS. In the multivariate analysis, five biomarkers remained independently associated with survival. NLR and SII demonstrated moderate discriminatory power (AUC > 0.70) for predicting the completion of three or more treatments.

**Conclusion:** Pretreatment inflammatory biomarkers are objective, readily accessible and clinically applicable tools that may support the selection of appropriate candidates for PIPAC. The findings of this study encourage the integration of biomarker assessments into future PIPAC research protocols.

## Introduction

Peritoneal metastasis (PM) is a common form of dissemination from various cancers. The severe symptoms associated with PM lead to a deterioration in the activities of daily living and affect the quality of life among patients 1-3. Treatment options are sparse, and most patients succumb to their disease within six months 1.

Pressurized Intraperitoneal Aerosol Chemotherapy (PIPAC) directed treatment has been introduced as a palliative alternative for patients with PM. PIPAC may lead to local control of PM, and selected studies have shown encouraging data on survival and quality of life 4-7. Contrary to the high degree of consensus regarding the technical details of PIPAC, uniform criteria for patient selection are lacking. Currently, patient selection for PIPAC is based on a combination of disease-related variables, such as previous treatment, ECOG Performance Status (PS), symptoms of bowel obstruction, ascites volume and extraperitoneal disease. The standard PIPAC regime comprises three treatment cycles, and their completion has been associated with improved survival outcomes 8. Nevertheless, a recent review of 53 studies involving 1990 patients reported that only 39% completed three or more PIPAC treatments, indicating that patient selection remains a significant challenge 9. This again emphasises the need for pretreatment prognostic tools that are objective, easily accessible and clinically applicable to support the selection of appropriate PIPAC candidates.

An ideal prognostic tool is non-invasive, easy to implement, cost-effective and standardised. Pretreatment blood tests exemplify such a tool, offering readily accessible data without additional clinical burden. Although tumour-specific biomarkers such as cancer antigen 125, carbohydrate antigen 19-9 and carcinoembryonic antigen are increasingly being used, their evaluation is complicated by the considerable heterogeneity observed in patient populations undergoing PIPAC directed therapy 10. In contrast, inflammatory biomarkers - routinely available as part of standard baseline blood testing - may offer a more viable alternative. An increasing body of evidence suggests that the host's systemic inflammatory response plays a central role in tumour development and progression 11, 12. Several inflammatory biomarkers have already demonstrated prognostic value across a wide range of malignancies, supporting their potential application in the context of PIPAC 13. Broadly, these biomarkers are categorised into cell-based inflammatory biomarkers and protein-derived inflammatory biomarkers.

### Cell-based inflammatory biomarkers

#### Neutrophil-to-Lymphocyte Ratio (NLR)

The NLR - reflecting the balance between innate (neutrophils) and adaptive (lymphocytes) immunity - is an established prognostic marker in cancer patients 14, 15. Several studies - including meta-analyses of patients with both operable and inoperable pancreatic, colorectal and gastric cancer - have reported that an elevated pretreatment NLR was significantly associated with reduced overall survival in multivariate analyses 14, 16. Three studies examined the prognostic value of the NLR in patients with PM 17-19. Two studies of patients with PM from pancreatic and colorectal cancer found an association between a high NLR and poor survival, whereas one study of patients with PM from colorectal cancer did not find the NLR to have an independent prognostic value 17-19.

#### Platelet-to-Lymphocyte Ratio (PLR)

The PLR reflects the balance between platelets and lymphocytes. Although less extensively investigated than NLR, evidence suggests its potential prognostic value. A meta-analysis of patients with locally advanced or metastatic cancer reported a significant association between elevated pretreatment PLR and reduced survival 20. The strongest association was seen in patients with renal cancer, but it was also observed in patients with gastrointestinal malignancies. Some trials lacked adjustment by multivariate analysis 20. One study of colorectal cancer patients with PM reported no association between an elevated PLR and survival 18.

#### Systemic Immune-Inflammation Index (SII)

This biomarker incorporates components of both the NLR and the PLR, since it is derived from neutrophil, lymphocyte and platelet counts. An elevated pretreatment SII in patients with gastric, colorectal and ovarian cancer has been significantly associated with a reduced overall survival 21-23. Of particular interest, a study of patients with PM from colorectal cancer found that the SII had a superior prognostic value compared to both the NLR and the PLR 18.

### Protein-derived inflammatory biomarkers

#### C-reactive protein (CRP)

CRP is a well-established and accessible marker of systemic inflammation with prognostic value in patients with incurable cancers, including those of gastrointestinal origin 24. A study on patients with metastatic colorectal cancer found that CRP was superior to other inflammatory markers in predicting survival 25. In addition, one study found that CRP was better than cell-based biomarkers in stratifying cancer patients into prognostic groups 13. Notably, no studies to date have examined the prognostic value of CRP specifically in patients with PM.

#### Modified Glasgow Prognostic Score (mGPS)

The mGPS is a protein-based inflammatory biomarker that combines measures of nutritional status (serum albumin) and systemic inflammation (CRP). Unlike other inflammatory biomarkers assessed on a continuous scale, mGPS employs a categorical scoring system (0, 1 or 2), offering a standardised and clinically applicable framework for prognostic evaluation 13, 26. Studies on patients with inoperable cancers - including patients with gastrointestinal or ovarian cancer - reported that patients with an mGPS above 0 had significantly poorer survival 26-29. In patients with PM from pancreatic cancer, mGPS has shown no significant prognostic value 19.

#### Prognostic Nutritional Index (PNI)

Like the mGPS, the PNI assesses both nutritional status and inflammation by a combination of serum albumin and lymphocyte levels. Of note, *low* PNI values indicate a poor prognosis. Originally developed for gastric cancer, the PNI has recently shown prognostic value across various cancer types, including pancreatic and colorectal cancer 30, 31. Importantly, a large study on PM of gastric origin (n = 660) found that a low PNI was linked to reduced overall survival in a multivariate analysis 32.

In summary, there is a need for pretreatment prognostic tools that are objective, easily accessible and clinically applicable to support the selection of appropriate PIPAC candidates. While inflammatory biomarkers have shown prognostic value in incurable abdominal cancers - including in some studies on patients with PM - their role in the context of PIPAC remains largely unexplored, with only one study to date 33. To address this gap, the primary aim of our study is to investigate the prognostic value of pretreatment, cell-based and protein-derived inflammatory biomarkers in patients with PM treated with PIPAC. Second, we explore the ability of these biomarkers to predict the completion of three or more PIPAC treatments.

## Materials and Methods

### Study design and participants

This observational study used data from patients with PM from gastric, pancreatic, colorectal or ovarian cancer, included in the prospective PIPAC-OPC1 and PIPAC-OPC2 studies at the Odense PIPAC Center (OPC) at Odense University Hospital in Denmark 5, 34. Detailed information on the inclusion and exclusion criteria, the PIPAC procedure, types and doses of chemotherapy and response assessment have been previously published 5, 34.

Patients were excluded from the study in cases of non-access to the abdomen during the first PIPAC procedure and from the specific biomarker analysis if baseline blood tests were obtained more than 21 days prior to the first PIPAC treatment.

This manuscript was prepared according to the Consolidated Standards of Reporting Trials (CONSORT) guidelines and fulfils the criteria of the Reporting Recommendations for Tumor Marker Prognostic Studies (REMARK) checklist 35. An artificial intelligence tool (ChatGPT-4) was used for text editing to improve clarity and readability in the introduction and discussion section.

### Baseline biomarker analysis

As described earlier, we investigated six different biomarkers of inflammation: three cell-based inflammatory biomarkers (NLR, PLR and SII) and three protein-derived inflammatory biomarkers (CRP, mGPS and PNI). Routine analyses of haematological parameters, albumin and CRP were performed in local laboratories. Haematological status included absolute lymphocyte, neutrophil and platelet counts, all measured in 10⁹/L. CRP and albumin levels were measured in mg/L and g/L, respectively. The calculations of NLR, PLR, SII and PNI, as well as the application of mGPS scores, were conducted blinded to patient characteristics and study outcomes, in accordance with the REMARK recommendations (Table 1).

### Outcomes

Survival was measured from the date of the first PIPAC until death from any cause. To address the number of treatments, a threshold of three or more PIPACs was used, thereby dividing the population into two specific groups.

### Statistics

Baseline characteristics were summarised for the overall population, the group receiving three or more PIPACs and the group receiving fewer than three PIPACs, using descriptive statistics. Categorical comparisons used Pearson's chi-squared or Fisher's exact test. The Kruskal-Wallis test was used for continuous variables. A p-value less than 0.05 was considered significant.

Survival analysis was conducted using Kaplan-Meier curves. Continuous biomarkers - that is, NLR, PLR, SII, CRP and PNI - were categorised into quartiles, and group differences were assessed by log-rank test. Cox regression was used for univariate and multivariate analyses, adjusting for covariates strictly available pretreatment (age, sex, PS, origin of primary tumour, primary tumour in situ, extraperitoneal dissemination, synchronous PM, time from PM diagnosis to the first PIPAC and number of palliative chemotherapy lines prior to PIPAC).

ROC curves reported the area under the curve, and ROC analysis determined optimal cut-off values at 95% specificity, with the corresponding positive predictive value and negative predictive value calculated.

Statistical analysis was performed using STATA® Software, Version 18 (Stata Corp, Texas, USA).

### Approvals and ethics

The PIPAC-OPC1 and PIPAC-OPC2 studies were conducted according to the Declaration of Helsinki, approved by the Regional Scientific Ethical Committee of Southern Denmark (Project IDs: S-20140211/S-20160100) and registered at www.clinicaltrials.gov (NCT02320448/NCT03287375). All participants were over 18 years of age and provided oral and written consent.

## Results

### Patient population

**Figure 1:**
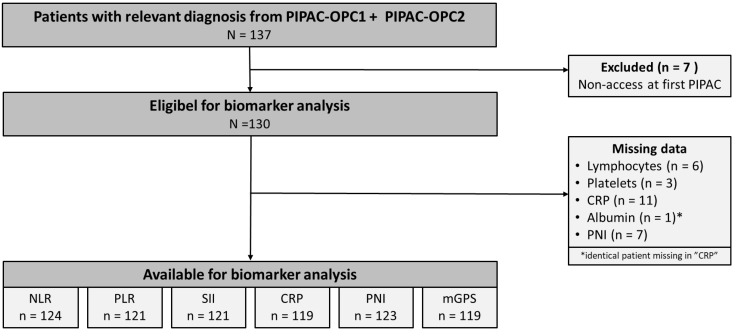
Consolidated Standards of Reporting Trials 2010 diagram presenting the patient flow in the study. CRP: C-reactive protein, mGPS: modified Glasgow Prognostic Score, NLR: Neutrophil-to-Lymphocyte Ratio, PIPAC: Pressurized Intraperitoneal Aerosol Chemotherapy, PLR: Platelet-to-Lymphocyte ratio, PNI: Prognostic Nutritional Index.

One Hundred Thirty-Seven patients with PM from gastric, pancreatic, colorectal or ovarian cancer were included in the PIPAC-OPC1 or PIPAC-OPC2 trials from March 2015 to January 2022. The last follow-up date was January 30, 2025, and data were extracted from the trial databases on January 31, 2025. Seven patients were excluded due to non-access at their first PIPAC, leaving 130 patients eligible for analysis. Biomarkers were missing in 6 patients (NLR), 9 patients (PLR and SII), 11 patients (CRP and mGPS) and 7 patients (PNI) due to blood samples taken more than 21 days prior to the first PIPAC, regional variations in blood testing or procedural errors. The patient flow is summarised in Figure 1.

#### Baseline characteristics

Table 2 presents the baseline characteristics of the total study population, the group receiving three or more PIPACs and the group receiving fewer than three PIPACs. There were no significant differences in the distribution of characteristics between the groups, except for *synchronous systemic chemotherapy* (p = 0.017) and the *volume of ascites at first PIPAC* (p = 0.002). A visualisation of the age distribution is depicted in the supplementary material (Figure S1) 35.

### Survival

**Figure 2:**
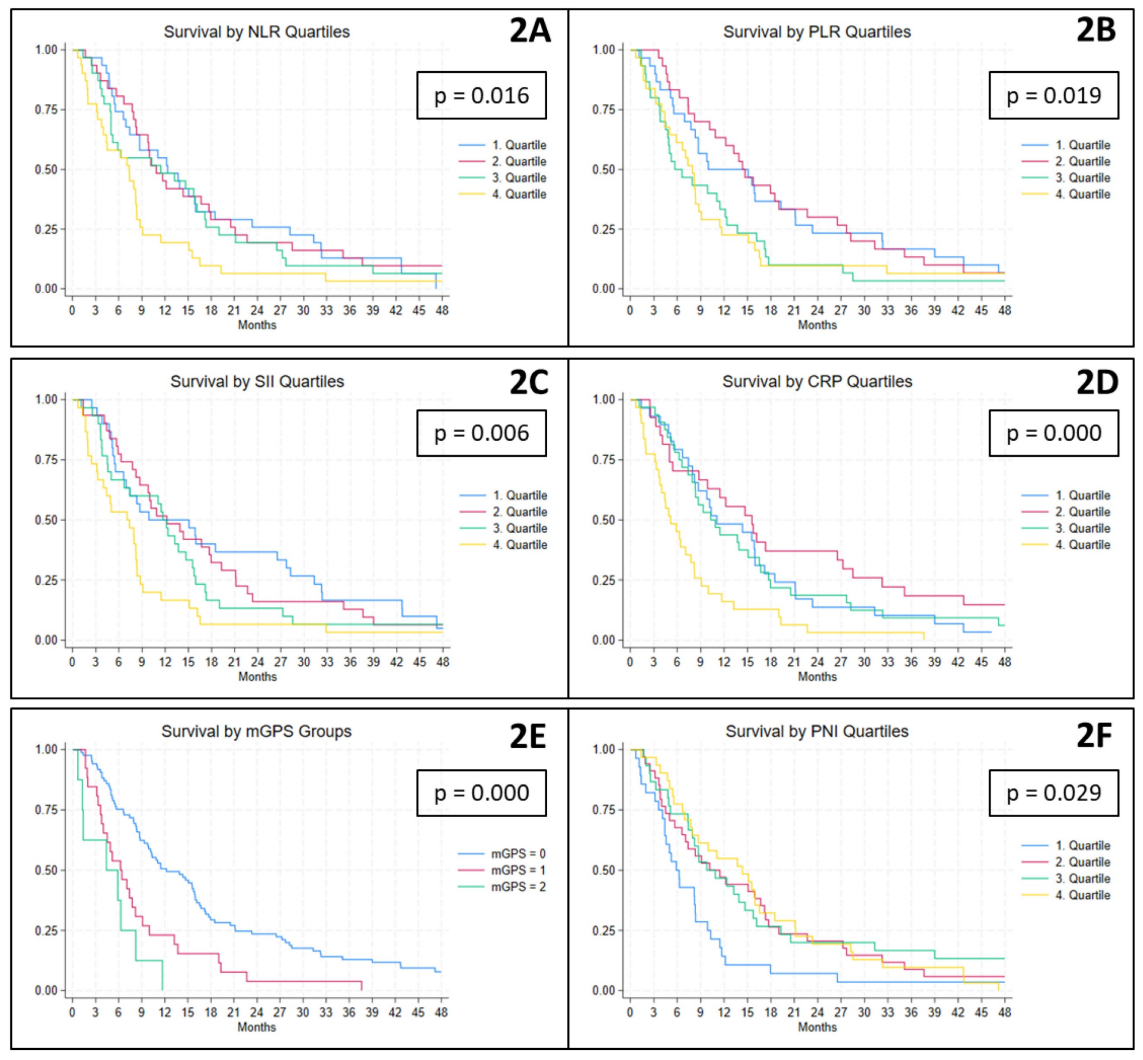
Kaplan-Meier plots illustrating the association between baseline biomarker levels and overall survival. NLR, PLR, SII, CRP and PNI are presented by quartiles, while mGPS is shown according to its predefined categories (scores 0, 1 and 2). CRP: C-reactive protein, mGPS: modified Glasgow Prognostic Score, NLR: Neutrophile-to-Lymhocyte Ratio, PLR: Platelet-to-Lymphocyte Ratio, PNI: Prognostic Nutritional Index, SII: Systemic Immune-Inflammation Index.

At the time of data extraction, 126 patients had died, and the median overall survival was 8.7 (IQR 4.9-16.5) months. In relation to *cell-based inflammatory biomarkers*, higher ratios were associated with poorer survival outcomes. Specifically, patients with baseline NLR and SII values in the fourth quartile had significantly shorter median survival times of 7.4 and 7.1 months, respectively, compared to 11.7 and 12.2 months among patients in the lower quartiles (Figure 2A and C). Similarly, for the PLR, patients with values above the second quartile experienced significantly reduced survival, with a median of 7.4 months, compared to 14.4 months in those below this threshold (Figure 2B). With regard to *protein-derived inflammatory biomarkers*, a comparable pattern was observed. Patients with CRP levels in the fourth quartile had a significantly shorter median survival of 5.2 months, as opposed to 11.5 months among the remaining patients (Figure 2D). For the mGPS, elevated scores of 1 and 2 were associated with significantly reduced survival, with medians of 6.2 and 4.4 months, respectively, compared to 12.2 months for patients with a score of 0 (Figure 2E). Finally, patients with PNI values in the first quartile had a significantly shorter median survival of 5.9 months compared to 11.5 months among those with higher PNI values (Figure 2F).

#### Regression analysis

The baseline values of all six biomarkers demonstrated prognostic value, with statistically significant hazard ratios (HRs) for death in the univariate analysis (Table 3). After adjustment for covariates, the cell-based inflammatory biomarkers NLR and SII remained statistically significant. Similarly, the protein-derived inflammatory biomarkers CRP, PNI and mGPS also retained significance. In addition, sex, PS and - specifically for the NLR - the origin of the primary tumour remained statistically significant. In both univariate and multivariate analyses, the continuous biomarkers were associated with HRs slightly above or below 1 (PNI). Although this may initially suggest limited prognostic value, it is important to acknowledge that these biomarkers are continuous variables with a broad range of values. Notably, the associated risk increases progressively with higher levels of these biomarkers, particularly at the upper end of the distribution.

### Predicting ≥ 3 or < 3 PIPACs

Of the 130 patients, 78 (60%) received three or more PIPAC treatments.

The pretreatment values of the six inflammatory biomarkers are presented in Table 4. Regarding the cell-based inflammatory biomarkers, both the median NLR and median SII were found to be significantly elevated in patients who received fewer than three PIPAC treatments compared to those who underwent three or more. With respect to the protein-derived inflammatory biomarkers, a statistically significant difference between the two groups was observed only for the mGPS.

**Figure 3:**
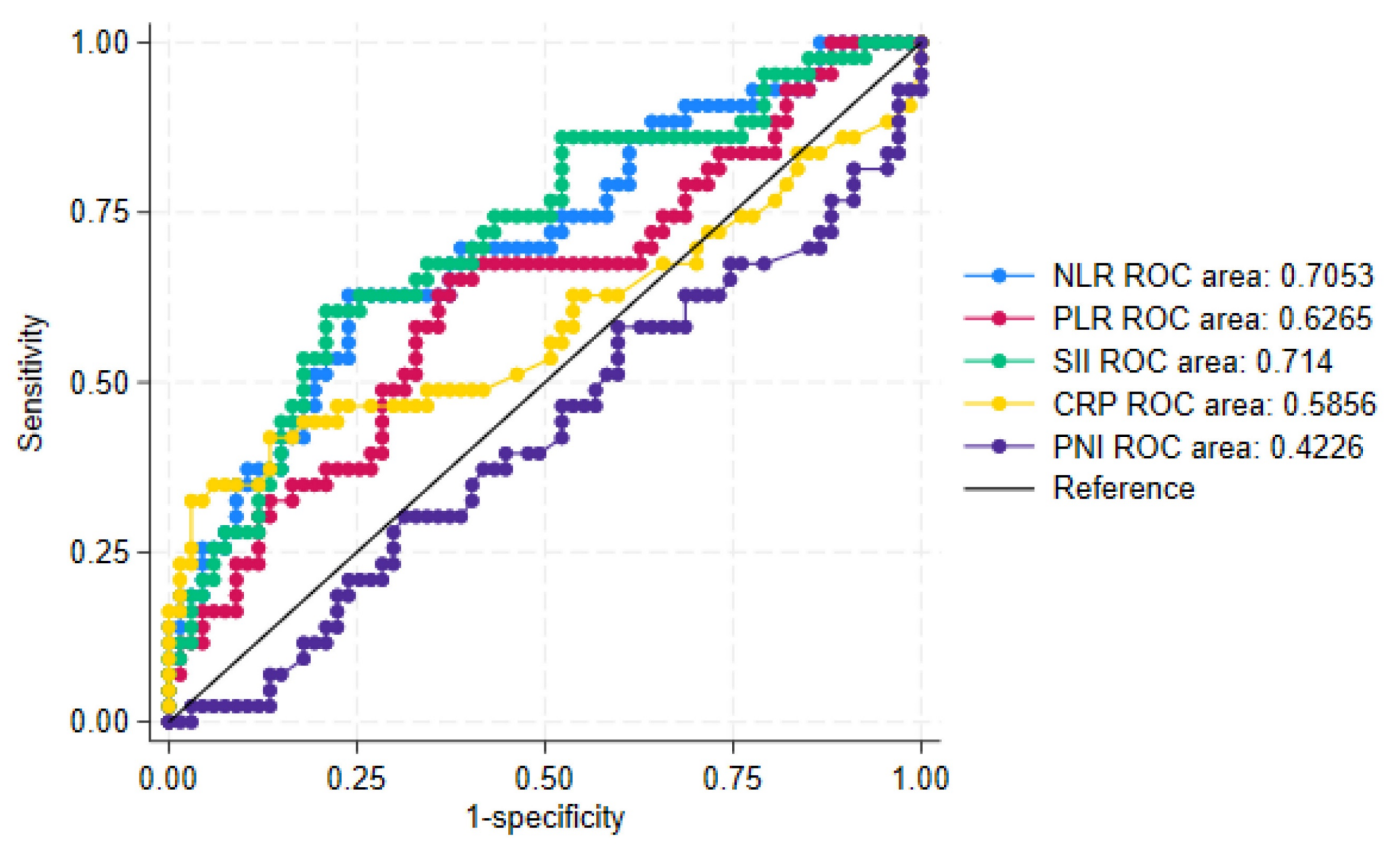
Figure of the combined ROC curves for the continuous biomarkers, including specific ROC areas. CRP: C-reactive protein, NLR: Neutrophile-to-Lymhocyte Ratio, PLR: Platelet-to-Lymphocyte Ratio, PNI: Prognostic Nutritional Index, SII: Systemic Immune-Inflammation Index.

Among the cell-based biomarkers, the SII demonstrated the highest discriminatory ability, with an area under the curve (AUC) of 0.714 (Figure 3). The NLR also showed acceptable discrimination, with an AUC of 0.7053. The PLR yielded a lower AUC of 0.6265. In contrast, none of the continuous protein-derived biomarkers exhibited sufficient discriminatory power to distinguish between patients receiving fewer than three compared to three or more PIPAC procedures.

The cut-off values at 95% specificity, along with their corresponding sensitivity and predictive values, are presented in the supplementary material (Table S1). Among the cell-based biomarkers, an NLR cut-off value of 5.140 and an SII cut-off value of 1667 yielded the highest positive predictive values (>80%) for identifying patients at risk of receiving fewer than three PIPAC treatments. Among the protein-derived biomarkers, CRP - with a cut-off value of 27 - returned a similar positive predictive value. However, values below the cut-offs were able to predict the chance of receiving three or more PIPAC treatments in only around 65% of the patients (the negative predictive value of the test).

A combination of the biomarkers SII and CRP did not result in predictive values that exceeded those mentioned above.

## Discussion

This study evaluated the prognostic relevance of six inflammatory biomarkers in a prospective cohort of patients with PM who were treated with PIPAC. We found that five of the six biomarkers - NLR, SII, CRP, mGPS and PNI - were independently associated with poorer overall survival. Second, the potential utility of these biomarkers in predicting which patients would receive fewer than three PIPAC treatments was explored. Among them, only the NLR and SII demonstrated acceptable discriminatory ability, with AUCs greater than 0.7. When applying cut-off values optimised for high test specificity, the NLR (≥ 5.140), the SII (≥ 1667) and CRP (≥ 27) demonstrated moderate ability to predict which patients would receive fewer than three PIPAC treatments in our population.

In general, these findings are in agreement with previous studies on the importance of inflammatory biomarkers in patients with incurable cancer, including gastrointestinal and ovarian cancer patients 14, 15, 26, 30, 36. To enhance comparability, this discussion focuses specifically on studies involving patients with PM.

### Cell-based inflammatory biomarkers

The prognostic value of pretreatment cell-based inflammatory biomarkers in patients with PM has previously been evaluated in three studies 17. Two of these focused on patients with colorectal PM undergoing cytoreductive surgery combined with either hyperthermic intraperitoneal chemotherapy or systemic chemotherapy. In line with the present findings, one study reported that a high NLR (≥ 3.1) was independently associated with reduced overall survival (HR 1.81) 17. In contrast, another study found no prognostic value for either the NLR or PLR; however, a high SII (≥ 410) was independently associated with poorer overall survival (HR 1.8), which supports the current results 18. A third study examined patients with PM of pancreatic origin undergoing various systemic treatment regimens and reported that a high NLR (≥ 5) was an independent prognostic indicator of worse overall survival (HR 1.68) 19.

Although these findings are broadly consistent with our results, several methodological and clinical differences must be acknowledged. First, all three studies reported homogeneous cohorts, whereas the present study included patients with heterogeneous primary tumours and in different lines of palliative treatment. Second, patients in the studies on PM of colorectal origin underwent curative-intent therapy, in contrast to the palliative treatment setting of our study, which may have influenced systemic inflammatory responses and survival outcomes. Third, all three studies employed dichotomisation of NLR, PLR and SII values - a methodological choice that may have inflated effect sizes and contributed to the discrepancies in the hazard ratios observed. Consequently, direct comparisons should be interpreted with caution.

### Protein-derived inflammatory biomarkers

The prognostic value of pretreatment protein-derived inflammatory biomarkers in patients with PM has been explored in three studies, two of which involved patients treated with modalities other than PIPAC and one focused specifically on PIPAC-directed therapy 19, 32, 33. One study investigating patients with PM of pancreatic origin found no prognostic benefit for those classified as mGPS 0, which contrasts with the present findings 19. However, it is important to note that - as in the current study - the subgroup of patients with an mGPS of 2 was small (3%), thereby limiting the ability to draw definitive conclusions.

A large study comprising 660 patients with PM of gastric origin treated with various palliative modalities reported that a low PNI was significantly and independently associated with reduced overall survival, lending support to the relevance of the PNI observed in the present study 32. Nevertheless, methodological differences should be considered. In particular, the study employed a dichotomous classification of PNI (< 45 vs. ≥ 45), yielding an HR of 0.81. This approach complicates direct comparison with the present findings, as our analysis treated biomarkers as continuous variables.

One study evaluated the prognostic value of the PNI in 51 patients who received PIPAC-directed therapy 33. They stratified patients into two groups by a predefined PNI cut off (< 36.5 vs. ≥ 36.5) and found that those in the group with the low PNI had a significantly reduced overall survival (HR 2.41). Again, a direct comparison to our results is hampered by this categorical approach. We chose to interpret PNI continuously because it preserves the full informational content of the data, enhances statistical power and enables the objective identification of optimal cut-off points. In contrast, categorisation can lead to information loss, reduced discriminatory ability and potential bias from arbitrary threshold selection 37. Although both study populations shared many similarities, baseline differences were apparent, with the median PNI considerably lower in this cohort (34.9 [26.2-42.0]) compared to that in our study (49.6 [43.5-53.0]), suggesting a higher degree of malnutrition in the former group.

Additionally, the study reported that a low PNI was strongly associated with a reduced likelihood of receiving multiple PIPAC treatments, achieving an outstanding AUC of 0.911. This finding contrasts substantially with our results, in which the PNI demonstrated the poorest performance in the ROC analysis, with an AUC below 0.5. Several methodological differences may account for these discrepancies, including differing thresholds for assessing the number of PIPAC treatments - 2 or fewer PIPACs compared to 3 or fewer in our study - as well as baseline nutritional disparities. Despite these divergences, it is noteworthy that the discriminatory ability of the NLR for predicting patients at risk of receiving fewer than the defined number of treatments was remarkably similar between studies, with both studies achieving an acceptable AUC of approximately 0.7. Comparative analysis of positive and negative predictive values was not possible, since the study did not report these metrics.

### Strengths and limitations

This study was strengthened by the inclusion of data from two prospective studies. Consequently, we had a large cohort with minimal loss to follow-up that contained 95% of the population available for biomarker analysis. It was also strengthened by strict inclusion and exclusion criteria, even though it is broadly impossible to have homogeneous study populations across different primary tumour types. On the other hand, selection bias and missing data may have influenced the results, and the heterogeneity of tumour origins may limit the generalisability of the findings. Additionally, variations in oncological treatment prior to or concomitant with PIPAC, as well as different intervals between treatments and blood sampling, could have impacted the biomarkers measured. A recent systematic review reported that less than 40% of patients had three or more PIPACs 9. The rate was 60% in the present study. Whether this represents a different selection of patients for PIPAC-directed therapy - and thus a (potential) limitation in the generalisability of our results - is difficult to assess. Finally, the use of local laboratories for blood testing has introduced potential data heterogeneity due to varying methods.

### Clinical implications and perspective

In the palliative treatment of patients with a dismal prognosis, such as those with PM, the guiding principle must be *should we treat?* rather than simply *can we treat?* In this context, biomarkers of inflammation represent objective, readily accessible and clinically applicable tools that may support the selection of appropriate PIPAC candidates. The findings of this study encourage the integration of biomarker assessment into future PIPAC research protocols. While the NLR remains a well-established inflammatory marker, the SII represents a potential alternative that warrants further exploration. Although CRP is relevant, its susceptibility to fluctuation suggests that it may be less reliable when considered in isolation 38. The mGPS offers a simple categorical framework that could enhance comparability across studies and support clinical interpretation.

Looking ahead, future studies should consider the potential value of serial biomarker measurements during and after treatment with PIPAC-directed therapy. For example, changes in inflammatory markers over time may signal when treatment should be discontinued, thereby providing real-time support for clinical decision-making. Moreover, comparative analyses between patients receiving PIPAC and those treated with systemic chemotherapy may help determine whether biomarker dynamics reflect treatment effects or underlying disease progression, particularly in the context of bidirectional treatment strategies. The use of tumour-specific biomarkers is hampered due to the heterogeneity of patients treated with PIPAC 10. A combination of inflammatory and tumour-specific biomarkers could be of interest.

In this study, cell-based and protein-derived inflammatory biomarkers were associated with overall survival in patients with PM treated with PIPAC. The NLR and SII also showed potential in predicting patients at risk of receiving fewer than three PIPAC treatments. Further research is needed to validate these findings and to determine their role in clinical decision-making.

## Supplementary Material

Supplementary figure and table.

## Figures and Tables

**Table 1:** Calculation of inflammatory biomarkers.

Inflammatory biomarker	Calculation
NLR	ANC/ALC
PLR	PLT/ALC
SII	(ANC x PLT)/ALC
PNI	Serum albumin (g/L) + (0.005 x ALC)
mGPS score	Definition
0	CRP ≤ 10 mg/L and albumin ≥ 35 g/L
1	CRP ≤ 10 mg/L and albumin < 35 g/L OR CRP > 10 mg/L and albumin ≥ 35 g/L
2	CRP > 10 mg/L and albumin < 35 g/L

ALC: absolute lymphocyte count, ANC: absolute neutrophil count, mGPS: modified Glasgow Prognostic Score, NLR: Neutrophil-to-Lymphocyte Ratio, PLR: Platelet-to-Lymphocyte Ratio, PLT: platelet count, PNI: Prognostic Nutritional Index, SII: Systemic Immune-Inflammation Index.

**Table 2:** Baseline demographics and PIPAC procedure related data of the total study population, the group receiving three or more PIPACs and the group receiving fewer than three PIPACs.

	Total (n = 130)	≥3 PIPACs (n = 78)	<3 PIPACs (n = 52)	p-value
**Age** (median, IQR)	63 (54-69)	64 (54-64)	62 (54-68)	0.460
**Sex** (n, %)				0.165
Male	52 (40)	35 (45)	17 (33)	
Female	78 (60)	43 (55)	35 (67)	
**ECOG PS** (n, %)				0.054
0	50 (38)	36 (46)	14 (27)	
1	77 (59)	40 (51)	37 (71)	
2	3 (2)	2 (3)	1 (2)	
**Primary tumour origin** (n, %)				0.394
Stomach	39 (30)	26 (33)	13 (25)	
Pancreas	26 (20)	15 (19)	11 (21)	
Colon	41 (32)	26 (33)	15 (29)	
Ovary	24 (18)	11 (14)	13 (25)	
**Primary tumour in situ** (n, %)				0.352
No	64 (49)	41 (53)	23 (44)	
Yes	66 (51)	37 (41)	29 (56)	
**Synchronous PM** (n, %)				0.713
No	50 (38)	29 (37)	21 (40)	
Yes	80 (62)	49 (63)	31 (60)	
**Extraperitoneal disease** (n, %)				0.052
No	111 (86)	70 (91)	41 (79)	
Yes	18 (14)	7 (9)	11 (21)	
**Previous lines of palliative chemotherapy** (n, %)				0.461
0	7 (5.4)	3 (3.8)	-	
1	77 (59.2)	50 (64.1)	4 (7.7)	
2	34 (26.2)	18 (23.1)	27 (51.9)	
3	8 (6.2)	5 (6.4)	16 (30.8)	
≥4	4 (3.2)	2 (2.6)	5 (9.6)	
**Time from PM diagnosis to PIPAC, months** (median, IQR)	7.3 (4.2-12.6)	7.0 (4.1-12.6)	7.8 (4.6-12.3)	0.572
**PROCEDURE-RELATED CHARACTERISTICS**				
**Number of PIPAC procedures**				-
1	29 (22)	0	29 (56)	
2	23 (18)	0	23 (44)	
3	39 (30)	39 (50)	0	
4	15 (11.5)	15 (19)	0	
5+	24 (18.5)	24 (31)	0	
**Synchronous systemic chemotherapy**				0.017
No	84 (65)	44 (56)	40 (77)	
Yes	46 (35)	34 (44)	12 (23)	
**ePIPAC**				0.567
No	108 (83)	66 (85)	42 (81)	
Yes	22 (17)	12 (15)	10 (19)	
**PRGS mean at first PIPAC**				0.061
≤2	56 (46)	39 (53)	17 (35)	
>2	66 (54)	35 (47)	31 (65)	
**Cytology at first PIPAC**				0.273
Negative	45 (37)	30 (41)	15 (31)	
Positive	76 (63)	43 (59)	33 (69)	
**PCI at first PIPAC** (median, IQR)	9 (3-19)	9 (2-17)	9 (3-22)	0.631
**Ascites at first PIPAC (mL)** (median, IQR)	10 (0-100)	0 (0-25)	40 (0-550)	0.002

ECOG PS: Eastern Cooperative Oncology Group Society performance status, ePIPAC: electrostatic precipitation Pressurized Intraperitoneal Aerosol Chemotherapy, IQR: interquartile range, mL: millilitre, PCI: Peritoneal Cancer Index, PIPAC: Pressurized Intraperitoneal Aerosol Chemotherapy, PM: peritoneal metastasis, PRGS: Peritoneal Regression Grading Score.

**Table 3:** Univariate and multivariate Cox regression analyses of inflammatory biomarkers and overall survival.

Variable	Univariate		Multivariate	
	HR	p-value	HR	p-value
COVARIATES				
Age (years)	1.005	0.613	-	-
Sex Female	1.8	0.002	-	< 0.05 (all biomarkers)
Performance Status 1 2	1.86 1.82	0.004	-	< 0.05 (all biomarkers)
Primary tumour origin		0.009	-	< 0.05 (NLR)
Gastric	reference			
Pancreas	0.87			
Colon	0.48			
Ovary	0.86			
Primary tumour in situ Yes	1.73	0.003	-	-
Synchronous PM Yes	1.38	0.081	-	-
Extraperitoneal disease Yes	1.17	0.545	-	-
Previous lines of palliative chemotherapy		0.631	-	-
1	1.04			
2	1.43			
3	1			
4	0.76			
5	1.66			
6	-			
7	1.17			
8	9.92			
Time from PM diagnosis to PIPAC 1 (months)	1.002	0.643	-	-
BIOMARKERS OF INFLAMMATION (range)				
Cell-based inflammatory biomarkers				
NLR (0.2-23.4)	1.14	0.002	1.13	0.003
PLR (36-721)	1.002	0.002	1.001	0.065
SII (53-9373)	1.0003	0.000	1.0003	0.000
Protein-derived inflammatory biomarkers				
CRP (0-318)	1.02	0.000	1.01	0.000
mGPS		0.0001		0.0005
1	2.2		1.8	
2	4.2		4.2	
PNI (25-64.3)	0.97	0.021	0.96	0.022

CRP: C-reactive protein, HR: hazard ratio, mGPS: modified Glasgow Prognostic Score, NLR: Neutrophile-to-Lymhocyte Ratio, PIPAC: Pressurized Intraperitoneal Aerosol Chemotherapy, PLR: Platelet-to-Lymphocyte Ratio, PM: peritoneal metastasis, PNI: Prognostic Nutritional Index, SII: Systemic Immune-Inflammation Index.

**Table 4:** Pretreatment values of the six inflammatory biomarkers.

Biomarker	Total study population (median, IQR)	≥ 3 PIPACs (median, IQR)	< 3 PIPACs (median, IQR)	p-value
Cell-based inflammatory biomarkers				
NLR	2.3 (1.5-3.3)	2.2 (1.3-3.0)	3.0 (1.8-4.1)	0.0009
PLR	146 (107-201)	135 (105-185)	159 (118-239)	0.054
SII	474 (360-839)	434 (339-567)	659 (437-1266)	0.0002
Protein-derived inflammatory biomarkers				
CRP*	4 (2-12)	4 (2-8)	4 (2-27)	0.1922
PNI	49.6 (43.5-53)	50 (43.7-53.3)	48.6 (42.1-52.3)	0.2116
	n (%)	n (%)	n (%)	
mGPS				0.013
0	85 (71)	n = 58 (80)	n = 27 (57)	
1	26 (22)	n = 12 (17)	n = 14 (30)	
2	8 (7)	n = 2 (3)	n = 6 (13)	

CRP: C-reactive protein, mGPS: modified Glasgow Prognostic Score, NLR: Neutrophile-to-Lymhocyte Ratio, PIPAC: Pressurized Intraperitoneal Aerosol Chemotherapy, PLR: Platelet-to-Lymphocyte Ratio, PNI: Prognostic Nutritional Index, SII: Systemic Immune-Inflammation Index. *Measured in mg/L.
