# Supplementary material for: The Role of Inflammatory Biomarkers in PIPAC: Predicting Survival and Treatment Completion in Patients with Peritoneal Metastasis
Source: J Cancer. 2026 Jan 1;17(1):10–20. doi: 10.7150/jca.123687 (PMC12719556; doi:10.7150/jca.123687)
Supplement: Supplementary file 1 — Supplementary figure and table. [file jcav17p0010s1.pdf]

Supplementary materials

Figure S1. Histogram depicting the age distribution in the total population.

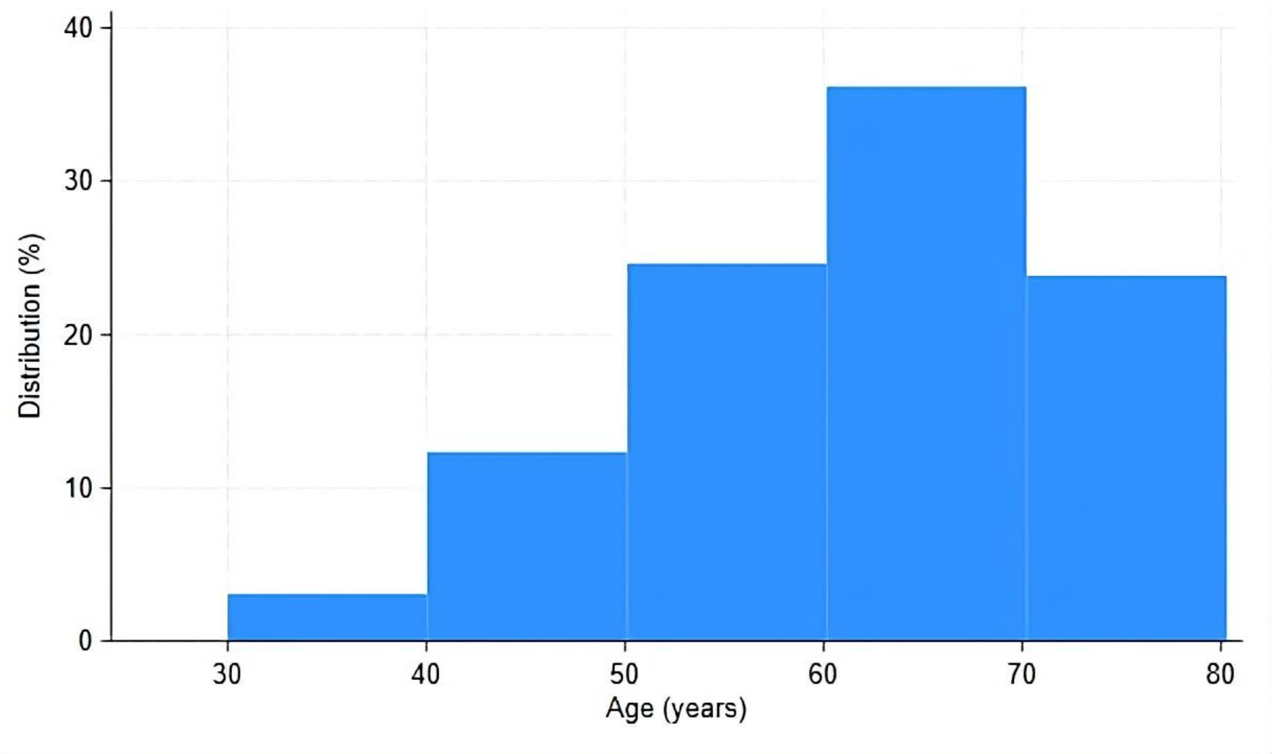

Table S1. Receiver operating characteristic analysis identifying cut-off values related to high test specificity.

Biomarker	Total (≥3 PIPACs/<3 PIPACs)	Cut-off	Sensitivity	Specificity	Positive predictive value (%)	Negative predictive value (%)
NLR	124 (75/49)					
		4.828	18.37	96.00	75	64
		**5.140**	18.37	97.33	**82**	65
		5.267	16.33	97.33	80	64
PLR	121 (73/48)					
		349.0	10.42	95.89	63	62
		350.4	10.42	97.26	71	62
		406.6	8.33	97.26	67	62
SII	121 (73/48)					
		1580	18.75	95.89	75	64
		**1667**	18.75	97.26	**82**	65
		1703	16.67	97.26	80	64
PNI	123 (74/49)					
		59.05	2.04	94.59	25	60
		59.5	2.04	95.95	25	60
		60.55	2.04	97.3	0	60
CRP	119 (72/47)					
		26	31.91	95.83	83	68
		**27**	25.53	97.22	**86**	67
		30	23.4	97.22	85	66
mGPS	119 (72/47)					
		0	-	-	-	-
		1	42.6	80.6	-	-
		2	12.77	97.2	75	63

The table displays associated sensitivity, specific specificity, and positive and negative predictive values. A positive predictive value is the probability of fewer than 3 PIPACs if the value of the biomarker is equal to or greater than the cut-off*. Negative predictive value is the probability of 3 or more PIPACs if the value of the biomarker is less than the cut-off*.

*conversely for the PNI.

CRP: C-reactive protein, mGPS: modified Glasgow Prognostic Score, NLR: Neutrophile-to-Lymhocyte Ratio, PIPAC: Pressurized Intraperitoneal Aerosol Chemotherapy, PLR: Platelet-to-Lymphocyte Ratio, PNI: Prognostic Nutritional Index, SII: Systemic Immune-Inflammation Index.
